# Progress in Studies of Surface Nanotextures and Coatings with Nanomaterials on Glass for Anti-Dust Functionality

**DOI:** 10.3390/nano12203677

**Published:** 2022-10-19

**Authors:** Liyong Wang, Mingming Liu, Yongling Wu, Hongyu Zheng

**Affiliations:** Centre for Advanced Laser Manufacturing (CALM), School of Mechanical Engineering, Shandong University of Technology, Zibo 255000, China

**Keywords:** anti-dust, easy cleaning, surface micro and nanotextures, superhydrophobic, anti-static

## Abstract

Dust pollution presents a wide range of adverse effects to product functionalities and the quality of human life. For instance, when dust particles deposit on solar photovoltaic panels, sunlight absorption is significantly reduced, and solar-to-electrical energy conversion yield may be lowered by 51%- Conventional (manual) dust removal methods are costly, consume significant material resources, and cause irreparable damage to the solar glass surface. Therefore, it is critical to develop glass surfaces that can clean themselves or are easily cleaned by natural forces. Many approaches have been attempted to reduce dust deposition, such as developing superhydrophobic surfaces and preparing anti-static surfaces. This paper reviews the recent progress in studies of anti-dust and cleaning mechanisms or methodologies, which include investigation into micro- and nano-sized dust properties, dust deposition processes and adhesion mechanisms to surfaces, and the state-of-the-art approaches to anti-dust and easy-cleaning functions that tailor surface micro-/nanotextures, lowering surface energy via nanocoatings, and enhancing anti-static properties with nanomaterials. We compare the advantages and disadvantages of various approaches and discuss the research prospects. We envision that future research will be focused on developing transparent surfaces with multiple dust-proof functions to cope with dust-burdening operating environments.

## 1. Introduction

Dust, which is found everywhere in life, often has a negative impact on agriculture, energy, healthcare, and transportation [1,2,3]. For instance, photovoltaic systems, which are expected to provide 3000 GW by 2030 [4], suffer from dust deposition on solar panels, causing a 70% reduction in efficiency in some Middle Eastern regions [5]. With the increasing level of industrialization, the global environment has been seriously polluted. The most obvious manifestations of this are water and air pollution. In large- and medium-sized cities, factories and cars emit a large volume of polluting soot during production and operation, which reacts chemically with water vapor in the air to produce acidic dust particles. Once the acidic dust particles adhere to product surfaces, they will cause severe corrosion and affect product functionalities [6]. Therefore, prevention of dust adhesion and generation of easy-clean surfaces have become a relevant and urgent need. Currently, contaminated surfaces are cleaned by active dust removal and passive dust removal techniques, which can be achieved using ultrasonic vibration, air jetting, mechanical wiping, laser cleaning, etc. [7,8,9]. However, such methods add additional costs and may damage the surfaces. Therefore, there is an urgent need to develop surfaces that rely on natural environmental conditions for easy cleaning.

In nature, there are many animals and plants that always keep their body surfaces clean and dust-free, such as lotus leaves [10], taro leaves [11], geckos [12], etc. This is because they have an exceptionally large number of micro-/nanotextures, and waxy layers with low surface free energy on their surfaces. Dust particles that fall onto these surfaces are easily carried away by rain or dew. Geckos can adhere to almost any surface, and in 2005, Hansen et al. [13] demonstrated that the bristles of geckos are an easy-cleaning adhesive, and that a gecko’s dusty feet need only a few steps to regain the ability to climb up a vertical wall. Inspired by nature, researchers have created various easy-cleaning surfaces [14,15].

The main reasons for dust particles being trapped on product surfaces are the various forms of adhesion forces, which include capillary forces, van der Waals forces, electrostatic forces, etc. [16,17]. A systematic study of dust particle properties can lead to better understanding of and solutions to dust adhesion issues. Researchers have found that dust properties vary greatly across regions. Lu et al. [5] studied four types of dust particles in different areas and found that the dust particles near coastal cities are significantly more humid than those further inland, and the dust particle size also varies significantly. Although the compositions are different, silicon dioxide (SiO_2_) and calcium carbonate (CaCO_3_) are found to be the most common components across different types of dust particles. Hassan et al. [18] found using CT scans that dust particles with large particle sizes generally have porous structures, while small-sized dust particles attach to the surface due to electrostatic adsorption [19]. Contact models simulating the natural settling process of dust particles and dust adhesion are important in providing a theoretical guidance for developing anti-dust surfaces.

**Figure 1:**
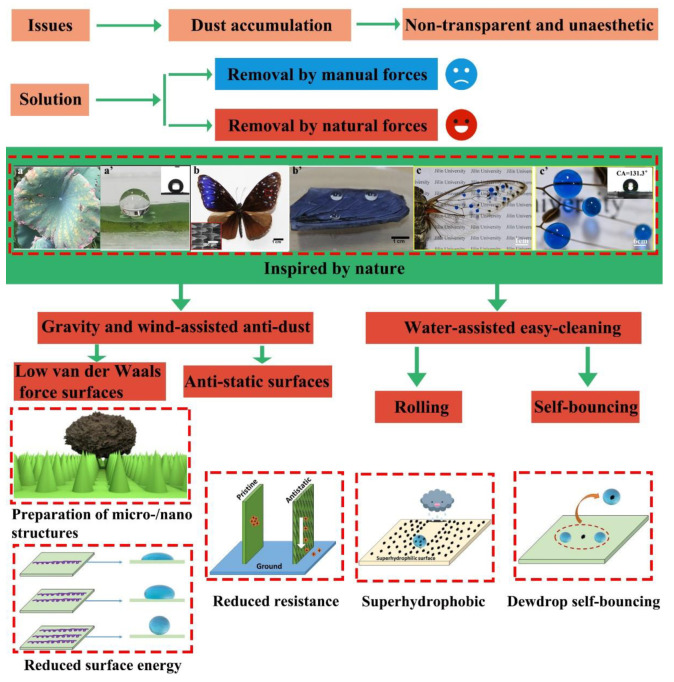
Illustration of the strategies for preventing dust accumulation and adhesion, and nature’s easy-cleaning phenomena. (**a**,**a’**) lotus leaf, reproduced with permission from Ref. [38]; (**b**,**b’**) butterfly wings, reproduced with permission from Ref. [39]; (**c**,**c’**) cicada wings, reproduced with permission from Ref. [40].

The main methods for producing dust-proof and easy-cleaning surfaces are: (a) preparing micro-/nanotextures on the substrate surface to reduce the contact area with dust particles [20,21,22]; (b) lowering the electrical resistance of the material surface to enhance its anti-static properties and reduce electrostatic attraction to charged dust particles [23,24,25]; (c) adding nano-sized active agents to the material surface to reduce the surface free energy and reduce the adhesion between the substrate and the dust particles [26,27,28]; (d) creating a superhydrophobic surface, which enables water droplets rolling across the surface to carry away dust particles [29,30,31]; (e) creating a superhydrophilic surface, which removes dust particles from the entire surface by generating a uniform water film on the substrate [32,33,34]; and (f) creating a photocatalytic surface, which decomposes organic dust particles on the surface by adding a photocatalyst to the coating [35,36,37]. Figure 1 shows an illustration of the strategies used to prevent dust accumulation and adhesion.

Considering the seriousness of dust deposition on glass products and the significance of developing low-cost, effective dust removal strategies, we aim to provide a comprehensive and in-depth review of anti-dust mechanisms and methods for the preparation of anti-dust surfaces using surface micro-/nanotextures and nanocoatings on glass surfaces. The review includes:

- The adhesion mechanism of dust particles on glass surface
- An overview of the various properties of dust particles, including composition, particle size, electrostatic charges, and the natural settling and adhesion processes of dust particles.
- Processes to create easy-cleaning surfaces under natural forces, including low van der Waals force surfaces, anti-static surfaces, and superhydrophobic surfaces.
- An analysis of the pros and cons of various dust prevention strategies, which include creating surface micro-/nanotextures, dust-repelling nanocoatings, and nanomaterials with anti-static properties.
- Future prospects in anti-dust research.

## 2. Adhesion Mechanism

Dust particles adhere to a wide range of glass surfaces with varying physical and chemical properties. The main reason for dust particles’ adhesion is the surface free energy. Solid surfaces reduce their surface energy by adsorbing tiny particles so as to bring them into a state of relative equilibrium. A force field exists around each mass in a solid, and since the arrangement of the masses inside the solid is ordered and repeated periodically, the force field of each mass is symmetric. However, at the surface of the solid, the periodic repetition of the mass arrangement is interrupted, so that the symmetry of the force field of the masses at the surface boundary is broken and the remaining bonding force is exhibited. As a result, the force balance is achieved by adsorbing tiny particles. The main forces between substrate surface and dust particles are classified as van der Waals, electrostatic, and capillary forces. Particle adsorption on a solid surface may be the result of one or several of these forces, which complicates the adhesion problem.

### 2.1. Van der Waals Forces

In a dry environment, capillary forces can be neglected [41,42]. Yilbas found that van der Waals forces are the main interfacial forces between dry dust particles and solid surfaces [43,44]. Based on rough surfaces, Rahinovich [45] proposed a new adhesion model, which can be expressed as:

(1) $$F_{vdw}=\frac{AR}{12D_{s}^{2}}\left(\frac{1}{1+\frac{R}{1.48rms}}+\frac{1}{{\left(1+\frac{1.48rms}{D_{s}}\right)}^{2}}\right)$$

where *R* is the radius of the dust particle, *D_s_* is the closest distance between the surface and the particle (approximately 0.4 nm), and *rms* is the root mean square of the asperity of the rough surface. *A* is the Hamaker constant, which is determined by the surface energy of the contact surface [46,47].

(2) $$A=24\pi D_{s\sqrt{\gamma_{p}\gamma_{s}}}^{2}$$

where γ_p_ and γ_s_ are the surface energies of the particle and wall surface, respectively. The van der Waals force decreases as the surface energies of the two contact surfaces decrease. In addition, coarsening the surface reduces the contact area between dust particles and the surface [45,48], which can substantially minimize the adhesion of dust particles. In conclusion, lower surface energy and coarser surface morphology will jointly reduce the van der Waals force.

### 2.2. Electrostatic Attraction

Static electricity is also a factor that affects the adhesion of dust particles to a glass surface, and atmospheric dust particles have a very complex charge problem [44]. According to statistics, 43% of the dust particles are not charged, 31% are positively charged, and the remaining 26% are negatively charged [44]. The electrostatic force includes the mirror image electrostatic force and the electrostatic contact potential, which is also called the double electric layer force. Two types of electrostatic forces can hold particles on a surface. The first type is generated by the presence of a large or excess charge on the surface, which attracts particles such as dust particles through Coulomb forces. For conductors, these excess charges leak through contact with other objects, so electrostatic adhesion forces are small. However, for non-conductors, the electrostatic attraction is significant. The mirror image electrostatic force is an important factor in dust adhesion to clothes and walls. As the ambient humidity increases, the appearance of a water film can allow the excess charge to leak out, so electrostatic attraction rarely occurs in a humid environment. The second type of electrostatic force is due to the contact potential caused by the difference in local energy states and electronic work functions when two different materials come into contact, and the contact potential causes a bilayer force to adsorb particles such as dust particles. The expressions for the mirror electrostatic force and the bilayer force are as follows [49]:

(3) $$F_{eI}=\frac{Q^{2}}{16\pi \in_{0}Z^{2}}$$

(4) $$F_{el}=\pi \in_{0}U^{2}$$

where *F_eI_* is the mirror image electrostatic force; *F_el_* is the double electric layer force; *Q* is the particle charged quantity; ∈_0_ is the dielectric polarization characteristic of the absolute dielectric constant; *Z* is the distance between the particle and the surface; and *U* is the contact potential difference.

### 2.3. Capillary Forces

In a humid environment, water vapor condenses in the space between two objects in contact, and particles are pulled toward the surface [50]. The capillary force is the additional pressure generated by the bending liquid surface, which is mainly due to the surface tension of the liquid. In general, mutual contact between the microparticles and solid interface surface morphology is not ideal, as it leads to a large number of microvoids or microcracks between the microparticles and contact surface. Such microvoids or microcracks would induce a capillary effect. When capillary forces exist, the magnitude of the capillary force plays a dominant role in the adhesion of dust particles to a substrate. When the air humidity is particularly high, it can account for 98% of the total adhesion force [44]. The formula for calculating the capillary force is as follows [51]:

(5) $$F_{c}=4\pi R\gamma \cos\theta$$

where *R* is the radius of the particle; γ is the specific surface energy; and θ is the contact angle of the liquid film between the particle and the surface. When the relative humidity (RH) is below 50%, the capillary force has no effect on the adhesion force. When the RH is in the range of 50 to 65%, the capillary force starts to take effect, and when the RH exceeds 65%, the capillary force dominates.

## 3. Properties of Dust Particles

**Figure 2:**
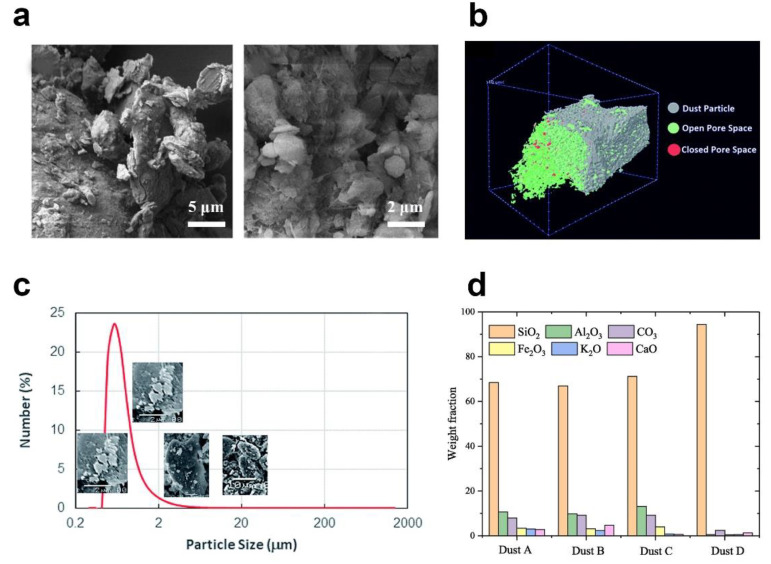
(**a**) SEM images of dust particles, reproduced with permission from Ref. [52]; (**b**) CT image of dust particles, reproduced with permission from Ref. [19]; (**c**) particle size distribution of dust particles, reproduced with permission from Ref. [18]; and (**d**) content and composition of dust particles, reproduced with permission from Ref. [5].

The study of dust particle properties is of great significant for dust prevention and dust removal. When observing particles under a scanning electron microscope (SEM), Abdelmagid et al. [52] found that sub-micron small dust particles were attracted to the larger micro-size dust particles or agglomerated with each other due to the electrostatic adsorption (Figure 2a). The presence of electrostatic charge is attributed to the fact that dust particles stay in the air for a long duration and interact with solar radiation. As shown in Figure 2b, the image of computed tomography indicates that the large dust particles present a porous structure, in which the open structure accounts for 20% of the total volume of dust particles [19]. Hassan et al. [18] analyzed the dust particles on the surface of photovoltaic panels in the area of Dammam and found that the size of the dust particles ranged from 1 μm to 10 μm, and the average diameter of dust particles was about 1.2 μm (Figure 2c). Lu et al. [5] studied four types of dust particles. The first type of dust particle was purchased from Powder Technology Inc, Arden Hills, MN, US, the second type was from Henan province in central China, and the third type was from Guangzhou city in southern China. The last type of dust particle was sand collected from construction sites. Figure 2d shows that among the four kinds of experimental dust particles, the most abundant components are SiO_2_ and Al_2_O_3_. Dhaouadi et al. [53] collected dust particles from parking lots in the United Arab Emirates, and through energy spectrum analysis, it was found that the contents of oxygen, carbon, aluminum, and silicon were in descending order, which were verified by XRD results. Table 1 lists the composition of the dust particles in different areas. Li et al. [6] summarized the specific surface energy of some dust particles. Different dust particles have different specific surface energy; for example, the specific surface energy of Al_2_O_3_ is 1.9 × 10^−4^ J/cm^2^, the specific surface energy of CaCO_3_ is 7 × 10^−6^ J/cm^2^, and the specific surface energy of MgO is 1 × 10^−4^ J/cm^2^. It can also be seen from Rahinovich’s surface energy formula [45] that the lower the surface energy of the dust particles and glass surface, the lower the adhesion between them.

Researchers have measured the charge of dust particles using a variety of methods [44,58,59], and the results show that dust particles in the Earth’s atmosphere have a charge of 6.3 × 10^6^ C/g for positively charged dust particles, and 7 × 10^6^ C/g for negatively charged dust particles. Merrison et al. [60] performed dust charging measurements using Mars analogue material. These dust particles were found to consist of negatively charged (46.6%) and positively charged (44.15%) particles. It was estimated that each dust particle typically carries a net charge of about 1.6 × 10^−14^ C. Normally, small dust particles are negatively charged, while large dust particles are positively charged [61]. Li et al. [62] compared indoor and outdoor dust particles and found that outdoor dust particles were affected by car emissions and construction, while indoor dust particles were affected by cooking, clothing, etc. Different types of dust particles adhere to glass substrate differently. Lu et al. [63] found that under the same experimental conditions, sand particles are easier to be removed than the soil particles.

**Figure 3:**
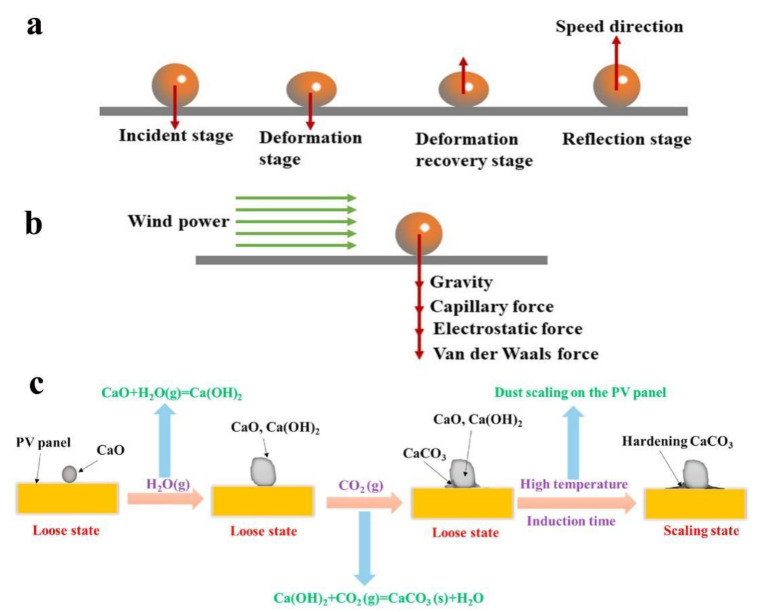
(**a**) Diagram of collisional adhesion of dust particles. (**b**) Adhesion and desorption of dust particles on a solid surface by external forces. (**c**) Schematic diagram of the scaling process of dust, reproduced with permission from Ref. [65].

Dust particles are affected by air convection and float with the air currents, and then gradually fall back to the ground under the force of gravity. As depicted in Figure 3a,b, dust and glass generally go through four phases when colliding: the incidence phase, deformation phase, deformation recovery phase and reflection phase [64]. If the adhesion force is greater than the rebound force, the dust particles will stay on the glass surface. For a short period, if the adhesion force can be overcome by an external force, the dust particles can be decoupled. But once they have stayed for a long time, the dust particles will change from loose to scaled state. Liu et al. [65] studied the scaling process of dust particles on PV glass and experimentally showed that calcium oxide can easily react with water vapor in the air to form calcium hydroxide. Subsequently, calcium hydroxide can interact with carbon dioxide to produce calcium carbonate (CaCO_3_). Although CaCO_3_ exhibits weak adhesion force in the initial stage, with an increase in temperature and humidity, CaCO_3_ begins to precipitate and gradually harden, exhibiting high adhesive strength on the substrate, which makes it extremely difficult to remove using natural forces (Figure 3c).

Lu et al. [66] studied the relationship between gravity and dust deposition using a hydrodynamic approach and found that gravity significantly affects the dust deposition rate, which can be 5% and 75% for small and large dust particles, respectively. Liu et al. [67] studied the law of particle accumulation governed by electrostatic forces and found that when airflow speed was increased, the weight of dust deposits was significantly reduced. This is consistent with the results of Lu [68]. Liu et al. [69] also explored the motion behavior and deposition mechanism of dust particles using the discrete element method, and the results showed that three situations occurred when dust collided with a surface. Firstly, the dust particles were directly adsorbed on the surface due to the high adhesion force. Secondly, the dust particles were ejected from the surface as a result of its own elasticity. The Poisson’s ratio of the dust particles was 0.4, the shear modulus was 2 × 10^6^, and the coefficient of recovery was 0.5. Thirdly, the dust particles slid or rolled on the surface for a certain distance before finally either detaching or staying on the surface.

## 4. Methods for Creating Anti-Dust and Easy-Cleaning Surfaces

Glass products such as displays, mirrors, and optical lenses play an important role in our daily work and life. However, these glass surfaces easily attract dust particles. In this section, we discuss methods for producing anti-dust and easy-cleaning surfaces via the generation of micro-/nanotextures, the application of low surface energy coatings, and the reduction in surface electrical resistance. Anti-dust surfaces need to be hydrophobic, which enables dust removal by gravity and wind due to weak van der Waals forces [47]. Anti-dust surfaces are particularly important in dry and rain-starved areas where easy-cleaning strategies relying on water do not work. Superhydrophobic easy-cleaning strategies that rely on water droplets to carry away dust particles can, however, work well in rainy areas [70,71].

### 4.1. Gravity- and Wind-Assisted Anti-Dust Surface

Anti-dust surfaces allow dust particles to be detached easily from the substrate surface by the effect of external forces. There are two main mechanisms for adherence between a substrate and dust particles: van der Waals forces and electrostatic forces [46,72]. It is crucial to develop anti-dust surfaces with low van der Waals forces, which result in a weak adhesion between a surface and dust particles. The dust particles can therefore be eliminated by external forces and do not rely exclusively on water droplets [73]. There are two ways to reduce van der Waals forces. The first is to build micro-/nanostructures to reduce the contact area between dust particles and glass substrates, and the other is to reduce the surface energy of the glass surface. Anti-static surfaces facilitate a reduction in dust deposition by weakening the electrostatic force between the dust particles and the surface [74].

#### 4.1.1. Low van der Waals Force Surfaces

**Figure 4:**
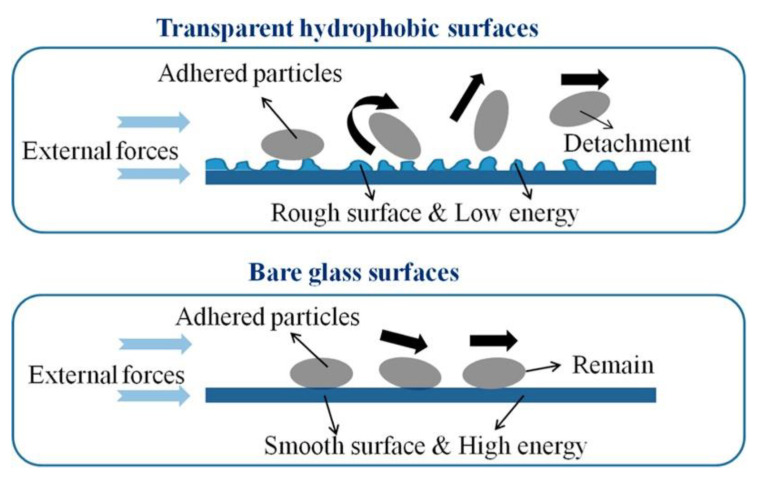
Comparison of dust removal from a superhydrophobic surface and a normal glass surface, reproduced with permission from Ref. [47].

The low surface energy coupled with the micro-/nanotextures reduces the adhesion between the dust particles and the surface. This type of anti-dust effect is less dependent on the strength of hydrophobicity. Experimental results showed that when the water contact angle was above 100°, the dust particles would be easily separated by gravity. In other words, both superhydrophobic and normal hydrophobic surfaces with the contact angle exceeding 100° would exhibit the anti-dust effect [75]. The schematic diagram in Figure 4 shows that a surface with a micro-/nanotexture and low surface free energy allows for easier dust removal by external forces as compared with smooth surfaces with high surface energy.

##### Surfaces with Micro- and Nanostructures

The existence of surface micro-/nanotextures, on one hand, can significantly decrease contact area with dust particles and thus weaken the adhesion between them and the surface [47,75]. Once the glass is tilted or subjected to external forces, the dust particles are easily dislodged. On the other hand, the micro-/nanostructure facilitates the rebound behavior of the dust particles, which is not conducive to dust deposition on the surface [76]. In the manufacture of photovoltaic panels, an anti-reflection coating is applied to reduce the surface reflection. The coating thickness is specially chosen so as to cause destructive interference and thus reduce reflection. The introduction of surface micro-/nanostructures would affect the glass transparency if the increased surface roughness is above a certain value. Larger scales of surface micro-/nanostructures lead to rougher surfaces, which would cause light scattering and reduce the transparency of the material. Different scales of roughness cause different scattering behaviors, mainly including Mie scattering and Rayleigh scattering [77,78]. When the surface roughness is on the visible wavelength scale, there is a high probability of significant light scattering by Mie scattering, and the surface will become opaque [79]. Assuming that the particles are all spherical, the total scattering cross section σ_M_ for Mie scattering is as follows:

(6) $$\sigma_{M}=\frac{\lambda^{2}}{2\pi }\sum_{m=1}^{\infty }\left(2m+1\right)\left({\left|a_{m}\right|}^{2}+{\left|b_{m}\right|}^{2}\right)$$

where *a_m_* and *b_m_* denote the Mie scattering coefficients, which are functions of particle radius r and refractive index n. The total scattering cross section σ_M_ increases exponentially with increasing particle size.

When the roughness is significantly smaller than the visible wavelength, the dominant light scattering is Rayleigh scattering [80], the formula for which is as follows:

(7) $$I=\frac{1+\cos^{2}\theta }{2S^{2}}{\left(\frac{2\pi }{\lambda }\right)}^{4}\left(\frac{n^{2}-1}{n^{2}+2}\right){\left(\frac{d}{2}\right)}^{6}\times I_{i}$$

where *θ* is the angle of incidence, *S* is the distance between the particle and the detector, *λ* is the wavelength of the incident light, *d* is the diameter of the surface roughness, *n* is the refractive index of the particle, and *I_i_* is the intensity of the incident light. The light scattering can be weakened by controlling the scale of the surface roughness structure below 70 nm [79].

There are several ways to construct micro- and nanostructures on a glass surface, such as the template method [81,82,83], laser ablation [84,85,86], chemical vapor deposition [87,88], electrospinning, [89,90], etc. However, there is a tendency for dust particles to become trapped in micron-sized structures. The desirable approach is by introducing a sol-gel coating using nanoparticles. This is because numerous studies have shown that particles smaller than 100 nm can be used to prepare structured surfaces to obtain superhydrophobicity and high transparency [63,91,92,93].

Silicon-based materials are widely used in anti-dust coatings because of their low cost and easy modification. Datta et al. [95] deposited 20 nm silica nanoparticles on a glass surface using a simple sol-gel method. The coating surface forms raised micro-/nanostructures. Wang et al. [96] dispersed nanosilica in butyl acetate solvent, then mixed it with fluorinated resin, polydimethylsiloxane, and ethanol, and finally formed a micro-/nanocolumnar structure on the glass surface by spraying. The 25 μm dust particles were used to simulate the natural dust particle falling process, and the experiment showed that the coating could greatly reduce the dust deposition on the glass surface.

**Figure 5:**
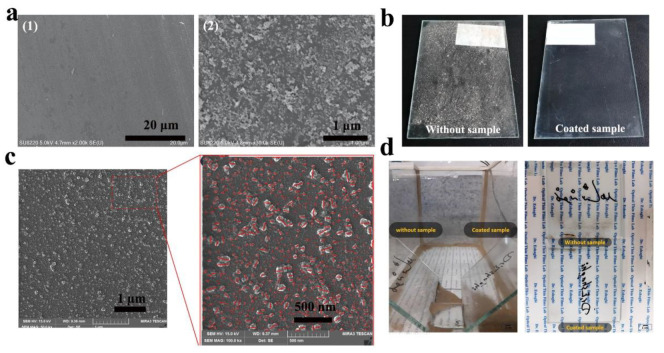
(**a**) (1) SEM image of a superhydrophobic surface; (2) A magnified view of (1) and (**b**) a comparative image of anti-dust properties, reproduced with permission from Ref. [94]. (**c**) SEM image of hydrophobic film and (**d**) comparison of anti-dust properties, reproduced with permission from Ref. [74].

Eren et al. [97] used the sol-gel method to construct a lotus-leaf-like textured structure on a glass surface and confirmed the long-term durability of the coating through testing. Zhang et al. [94] mixed silicone resin, ethanol, and silica nanoparticles to form a superhydrophobic coating, which was uniformly applied to the glass (Figure 5a). Compared with the uncoated bare glass, the coated glass surface possessed a micro-/nanostructure and thus exhibited excellent dust resistance in anti-dust testing (Figure 5b). Salehi et al. [74] deposited a silica film modified with hexamethyldisilane and PDMS on a glass substrate using a spray method, with 10–70 nm silica nanoparticles uniformly distributed on the surface of the film (Figure 5c). In the dust test, the deposition of dust particles on the coated glass surface was significantly reduced. (Figure 5d).

**Figure 6:**
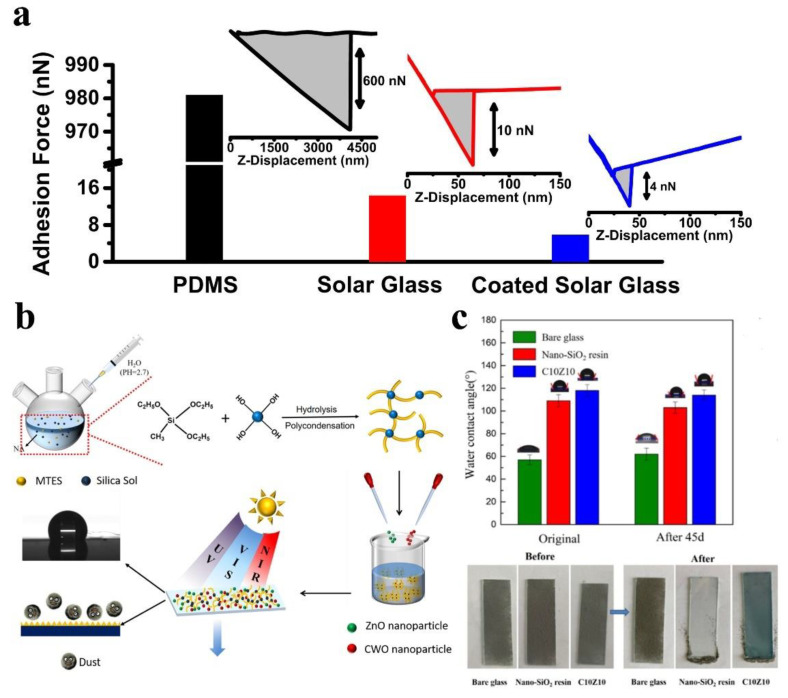
(**a**) Measurement of adhesion between dust particle and surface, reproduced with permission from Ref. [98]. (**b**) Preparation process and (**c**) anti-dust properties of anti-dust coating, reproduced with permission from Ref. [99].

In order to structure the surface and minimize the contact zone between the dust particles and the glass, Polizos et al. [98] prepared a silica coating with a nanostructured surface that reduced the adherence between dust particles and substrate. Adhesion measurement results revealed that a force of 10 µN was needed to remove 15 µm silica particles from an uncoated glass surface, but a force of only 4 µN was needed to remove them from a coated glass surface. The addition of the coating therefore significantly reduced the adherence (Figure 6a). The results of anti-dust tests showed that coated glass can increase light transmission by 20% relative to bare glass in the UV-visible range. To investigate whether the micro-/nano-structure or the surface energy played a major role in dust particles repellency, Pan et al. [76] constructed a micro-/nanostructure by dissolving silica in alcohol and spraying it on the glass surface, then prepared a low-surface-energy silica sol using the sol-gel method and combined the nanosilica with silica sol to prepare a superhydrophobic surface. The experiments showed that a micro-/nanostructure can significantly increase dust particle rebound and reduce the dust deposition compared to low surface energy only, and the combination of a micro-/nanostructure with a low energy surface can effectively repel dust particles. Li et al. [99] prepared an anti-dust and anti-fog nanostructured coating using a sol-gel preparation process, which is schematically shown in Figure 6b. The contact angle of the anti-dust coating was less than 150°, but still maintained an excellent dust particle repellent effect (Figure 6c).

**Figure 7:**
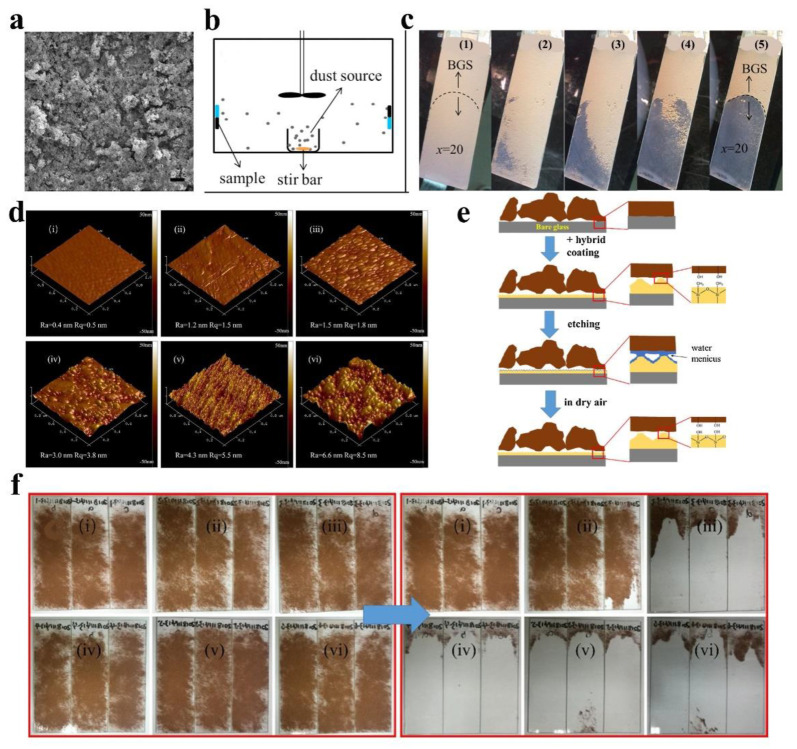
(**a**) SEM image of silicon-based hydrophobic coating. (**b**) Anti-dust properties test device. (**c**) Dust particles are blown away from the coating surface by the wind, reproduced with permission from Ref. [47]. (**d**) Morphology diagram and (**e**) anti-dust mechanism of transparent hydrophobic coating. (**f**) Anti-dust performance of hydrophobic coating under gravity, reproduced with permission from Ref. [75].

Dust particles are generally removed by the influence of wind and gravity on anti-dust surfaces. Quan et al. [47] verified the dust-proof performance of hydrophobic coatings by wind removal. The coatings are composed of SiO_2_ and silica sol, as shown in Figure 7a. Micro-/nanostructures were distributed across the coating surface. Figure 7b shows a homemade anti-dust test chamber with dust particles lifted by stir bar and fan. Half of the glass sample is coated, leaving the other half as bare glass. Figure 7c demonstrates the gradual detachment of the dust on the coated side under the effects of the wind. However, the dust particles on the bare glass surface (BGS) were not removed. Zhang et al. [75] verified the anti-dust performance of coatings by gravity removal, as depicted in Figure 7d. With an increase in the ratio of SiO_2_ to silica sol from ⅰ to ⅵ, the morphology of the coating surface changes from flat to rough. Figure 7e shows that micro- and nanostructures can effectively reduce the contact point between dust particles and coating surface and reduce the adhesion. In addition, the hydrophobic methyl group reduces the surface energy of the coating and therefore reduces the deposition of dust particles. Figure 7f shows that under the action of gravity, ⅰ and ⅱ coatings without micro-/nanostructures hardly have the function of anti-dust, while ⅲ to ⅵ show excellent anti-dust performance, which further verifies the important role of surface micro-/nanostructures.

Similarly, titanium dioxide [100,101], zinc oxide [102,103], alumina [104,105], and other nanoparticles can also be used to construct micro-/nanostructures. Wang et al. [100] used TiO_2_ microspheres mixed with epoxy resin and applied to a glass surface. Uniformly distributed nanosheets were grown on the surface of TiO_2_ microspheres, which made the coating with a micro-/nanostructure and turned into a superhydrophobic surface. Mayengbam et al. [106] used swept-angle deposition to grow vertically aligned zinc oxide (ZnO) nanowires that can reach a contact angle of 126° without hydrophobic treatment, showing great potential for dust-proof and easy-cleaning surfaces. Xu et al. [91] grew ZnO nanorods with a diameter of approximately 27 nm on photovoltaic panels, and the contact zone of dust particles with the coated surface was significantly reduced compared with that of ordinary glass. After surface modification with fluoride, the nanocrystal composite coating could achieve 80% removal efficiency for dust particles of 50–100 μm in size. Sutha et al. [1] coated glass with alumina sol, annealed it in a high temperature furnace at 400 °C for 60 min, and finally immersed it in hot water for 20 min to obtain porous alumina films. The coating had a static contact angle of 161° and exhibited superior easy-cleaning properties at inclination angles of less than 10°.

##### Low Surface Energy Surface

Anti-dust coatings are prepared by adding an appropriate surfactant to the basic formulation coating to further reduce the surface free energy of the substrate. The coated surface can weaken the interfacial adhesion between dust and surface and allow dust particles to be easily removed at low fluid shear [26], thus obtaining anti-dust properties. Among the known materials, fluorinated materials are considered to have the lowest surface energy; therefore, the addition of fluorinated materials can effectively reduce the surface energy [98,107].

**Figure 8:**
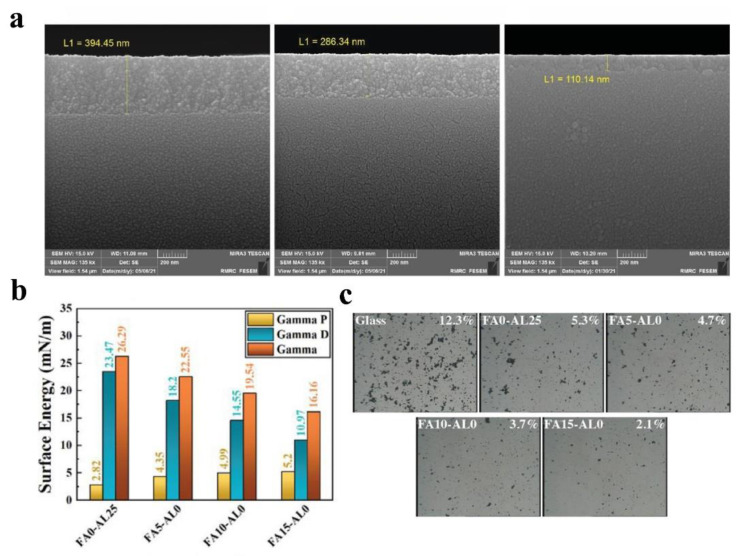
(**a**) SEM images of the cross-section of the coated glass. (**b**) Surface energy of the coating and (**c**) optical micrographs after dust-fall experiments, reproduced with permission from Ref. [107].

To explore the effect of surface energy alone on anti-dust properties, Taheri et al. [107] synthesized a set of hydrophobic coatings with controlled surface chemistry using fluoroalkylsilanes. As shown in Figure 8a, the surface of the coatings is extremely flat with almost no micro-/nanostructure, and the roughness can be kept below 1 nm. As the amount of fluoroalkyl silane increases, the surface energy gradually decreases (Figure 8b). As a result, the dust particles are also gradually reduced, as shown in Figure 8c. However, fluorine is toxic and environmentally unfriendly, and in the spirit of green and sustainable development, the use of non-fluorinated silanes to modify the surface is more strongly advocated. Therefore, recent research has been focused on using fluorine-free silica sol to prepare anti-dust surfaces. For example, some fluorine-free silica sol coatings include methyltriethoxysilane [75], hexamethyldisilazane [47,63,74], and polydimethylsiloxane [74]. Pan et al. [76] found that the dust deposition on the surface of glass coated with hydrophobic silica sol was only 51.4% of that of the bare glass, which proves that the low surface free energy property plays an essential role in anti-dust performance.

#### 4.1.2. Low Electrical Resistance Surface

Airborne dust particles are generally charged, so they can easily be firmly attached to the surface of glass substrates due to electrostatic forces. A common strategy is to prevent the accumulation of static electricity by lowering the surface electrical resistance to leak the charge out, thus minimizing dust deposition. This is because sheet resistance between 10^5^ Ω/Sq and 10^12^ Ω/Sq usually allows the charge to disappear in a few milliseconds [23] to avoid dust deposition. The way to reduce the surface electrical resistance is to add some anti-static agents. In a dry atmosphere, static electricity can easily lead to the adsorption of dust particles. In addition, when the dust particles collide with the insulated glass surface, static electricity will be generated, and the charge is also difficult to predict [108]. Therefore, preparing a layer of anti-static film on the glass can decrease the surface electrical resistance and reduce the adhesion of dust particles.

**Figure 9:**
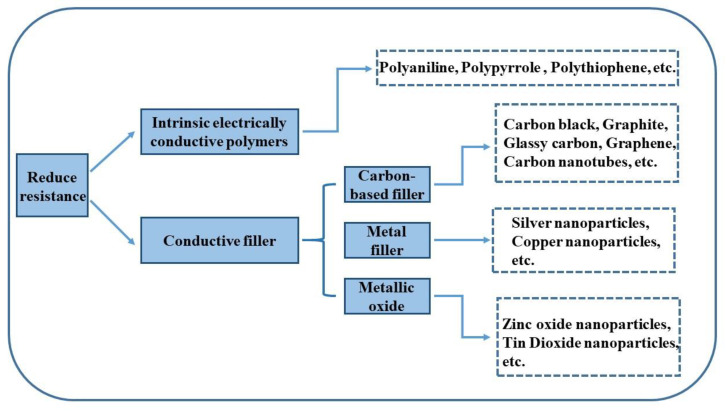
Strategies to reduce surface electrical resistance.

Dong et al. [109] prepared a SnO_2_-SiO_2_ coating using the sol-gel method. SnO_2_ has high electrical conductivity, which helps to lower the coating sheet resistance to 6.95 × 10^6^ Ω/sq in the anti-static range. With higher SnO_2_ content, the coating has a better ability to remove 50% of the dust particles. Fenero et al. [23] added conductive laponite to the coating, reducing the sheet resistance to 10^7^ Ω/sq. These experiments showed that the coating could repel dust particles effectively [24]. Figure 9 provides an overview of several common methods of reducing surface sheet resistance, such as by the addition of intrinsically conductive polymers [110], metal particles [111], metal oxides [112], and carbon-based 2D materials [113,114]. Ma et al. [114] laminated graphene, epoxy resin and finally obtained a transparent conductive film with a transparency of 96.7% and a surface sheet resistance of about 146 Ω/sq.

### 4.2. Water-Assisted Easy-Cleaning Surface

#### 4.2.1. Dust Removal Using the Motion of Water Droplets

Superhydrophobic surfaces have considerable potential for applications in taking away dust particles using water droplets, owing to their low surface free energy [115,116] and rough micro-/nanotexture [117]. The hydrophobicity of lotus leaves is caused by the micro-/nanostructure and waxy layer on the surface [118,119]. Inspired by this, many scholars have prepared superhydrophobic surfaces using various techniques [120,121,122]. The hydrophobicity reduces the friction between water and the glass surface, making it easier for water droplets to roll along the surface, while carrying away the dust particles [94]. Heckenthaler et al. [123] found that dust particles are attracted to the water-air interface and removed from the surface as droplets move along it. Hydrophobic modifications or structuring the surface can increase the ability to remove particles through easy-cleaning mechanisms. Hydrophobic and superhydrophobic surfaces remove dust particles in different ways. On hydrophobic surfaces, hemispherical droplets carry away dust particles by sliding, and on superhydrophobic surfaces, spherical droplets carry away dust particles by rolling. Parkin et al. [124] found that rolling droplets are more effective in picking up dust particles than sliding droplets.

**Figure 10:**
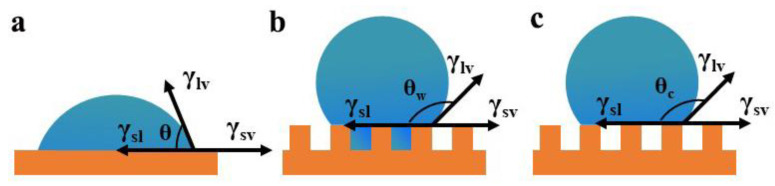
(**a**) Young’s contact angle, (**b**) Wenzel model, and (**c**) Cassie-Baxter mode.

Wetting properties are mainly dependent on the chemical properties and microstructures of the surface. Based on the water contact angle (WCA) of the surface, wettability can be classified as superhydrophilic surface, hydrophilic surface, hydrophobic surface, and superhydrophobic surface surfaces [125]. As shown in Figure 10, liquid contact models are generally classified as the Young’s contact angle model [126], Wenzel model [127], and Cassie-Baxter mode [128]. The Young model’s equation treats the surface as ideally smooth, while the actual solid surface has a rough structure. The Wenzel model uses a roughness factor to modify the Young model’s equation. However, the Wenzel model cannot explain the superhydrophobic phenomenon. Thus, Cassie and Baxter et al. made further modifications to the Wenzel model.

Techniques for preparing superhydrophobicity on glass surfaces are divided into top-down and bottom-up processes. The top-down approach involves the preparation of nanostructures of the desired size from larger substances using various etching techniques. Bottom-up approaches refer to the self-assembly of a number of simple, smaller units into relatively large and more complex systems. Top-down processes include laser machining, plasma etching, etc. Bottom-up approaches include electrostatic spinning, sol-gel coating, etc. Table 2 summarizes the common methods for constructing superhydrophobic surfaces.

**Figure 11:**
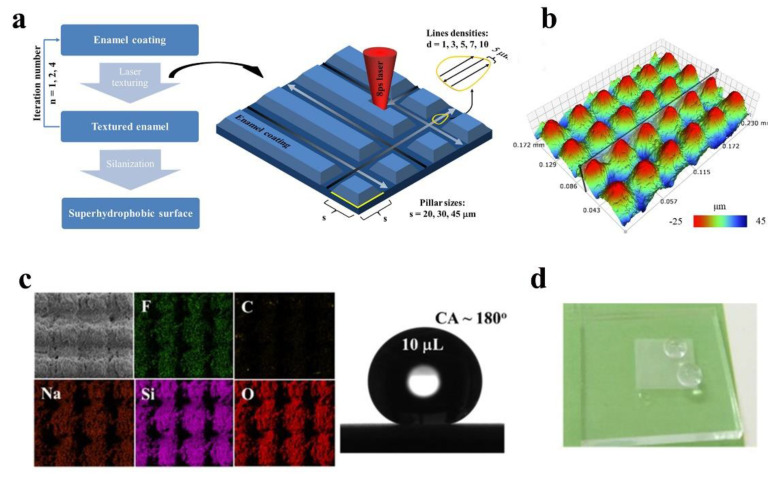
(**a**) Schematic diagram of laser etching, (**b**) 3D structural morphology of the glass surface. (**c**) Element distribution and contact angle of the glass surface, reproduced with permission from Ref. [129]. (**d**) The superhydrophobic surface after laser machining, reproduced with permission from Ref. [148].

In the top-down approach, laser machining is an efficient and maskless microfabrication technique that allows the preparation of layered structures on a variety of materials to produce superhydrophobicity [143,144,145]. The most common lasers are nanosecond [146], picosecond [129], and femtosecond lasers [147]. The biggest advantage of lasers is their high precision, which can accurately write micro- and nanostructures directly on the glass surface and maintain high visible light transmittance. Nguyen et al. [129] constructed conical columnar structures on inorganic glass using a laser and subsequently treated the surface with low surface energy perfluorooctyltriethoxysilane. Figure 11a shows the schematic diagram of laser processing, and Figure 11b shows the morphology after laser processing. These micro-/nanostructures resemble the structures on the surface of a lotus leaf. The measured contact angles were nearly 180°. Wang et al. [148] successfully constructed micron-scale groove structures on glass surfaces by picosecond laser, using the low surface energy substance 1H, 1H, 2H, 2H-perfluorodecyltriethoxysilane for hydrophobic modification. The contact angle of the modified surface was up to 172°. Figure 11d demonstrates the superhydrophobicity of the glass surface.

**Figure 12:**
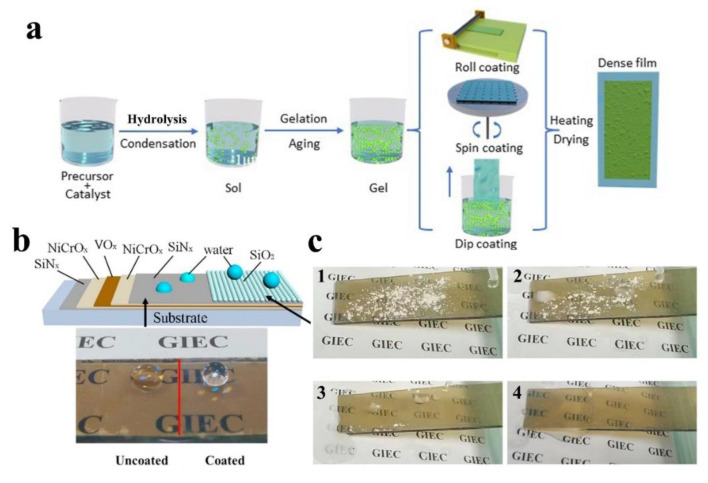
(**a**) Schematic diagram of the preparation of sol-gel. (**b**) Schematic of multi-layer structure coating. (**c**) Easy-cleaning property of the composite films, reproduced with permission from Ref. [151].

In the bottom-up approach, the sol-gel process is efficient and cost effective. Figure 12a shows the preparation process of sol-gel, where the precursors are subjected to hydrolysis and condensation reactions, and then, after a certain aging time, a transparent coating is formed on the glass surface by roll-on, spin-on and lift-off methods [136,149,150]. Sol-gel does not require expensive instruments and the cost of raw materials is low, which is suitable for large area production and is now widely used in the preparation of glass coatings.

As depicted in Figure 12b, Cong et al. [151] used magnetron sputtering and sol-gel to prepare smart glasses with superhydrophobic and thermochromic properties, with water contact angles up to 165°. The coating exhibits superior easy-cleaning properties, and with the assistance of water, dust particles on the glass surface are easily washed away (Figure 12c). Mahadik et al. [152] developed a simple and low-cost method to create multifunctional superhydrophobic coatings by depositing a modified silica layer on a glass substrate. The coating is transparent and has a WCA of 153°. Durability is a key factor in whether superhydrophobic coatings can be used in real-life applications. To address this issue, Hashjin et al. [153] prepared superhydrophobic coatings with 160° contact angle using nanosilica to construct a rough surface, and triethoxyoctylsilane as a low surface energy precursor. The robustness of the coatings could be significantly improved by alternately spraying sols of different pH values on the glass. Durability tests showed a lifetime of 1500 h for the superhydrophobic behavior.

Like superhydrophobic surfaces, superhydrophilic surfaces are also easy to clean, as they remove dust particles from the entire surface by forming a uniform water film on the solid surface [32]. Nature’s fish scales, shark skin, and forest frogs have superhydrophilic properties [154,155]. Inspired by nature, researchers realized two key factors for achieving superhydrophilicity: the high-energy surface and the rough surface structure. Lu et al. [156] prepared superhydrophilic silica films on glass surfaces. The transparent, superhydrophilic films consisted of silica nanoparticles with diameters of about 20–30 nm. Tao et al. [157] constructed super hydrophilic multifunctional coatings based on SiO_2_ and TiO_2_ nanomaterials, which can reach an average light transmission rate of 97.7, with both self-cleaning and anti-reflective effects. Lei et al. [158] modified α-zirconium phosphate nanoplates to obtain a highly transparent superhydrophilic glass coating. The results showed that the contact angle of the coating could approach 0°. Lu et al. [159] mixed the prepared TiO_2_ and SiO_2_ sols and stirred them continuously for 4 h. Finally, the sols uniformly coated the bare glass samples, and the contact angle could reach 5°. After the water spray treatment, the remaining dust particles’ mass ratio of the superhydrophilic surface was 18.6%, which indicated the excellent cleaning function of the coating. Bakri et al. [160] sprayed a new nanomaterial based on TiO_2_ on the surface of solar glass and obtained superhydrophilic and photocatalytic surfaces, which showed excellent dust-proof performance in three months of outdoor experiments.

#### 4.2.2. Dust Removal Using Dewdrop’s Self-Bounce

In addition to the rolling of water droplets taking away dust particles, researchers have found that the self-bouncing of water droplets can also carry away dust particles. Superhydrophobic surfaces have excellent easy-cleaning properties, which are mainly reflected in the large amount of dust particles carried away by external forces or when water droplets roll. However, in some dry regions, there is often a large temperature difference between day and night, accompanied by high air humidity, so condensation becomes an important means of dust removal. Several studies have investigated the self-bouncing effect and the cleaning effect of condensation on a superhydrophobic surface [161,162]. For superhydrophobic surfaces, the surface energy released by water droplet binding leads to bouncing.

**Figure 13:**
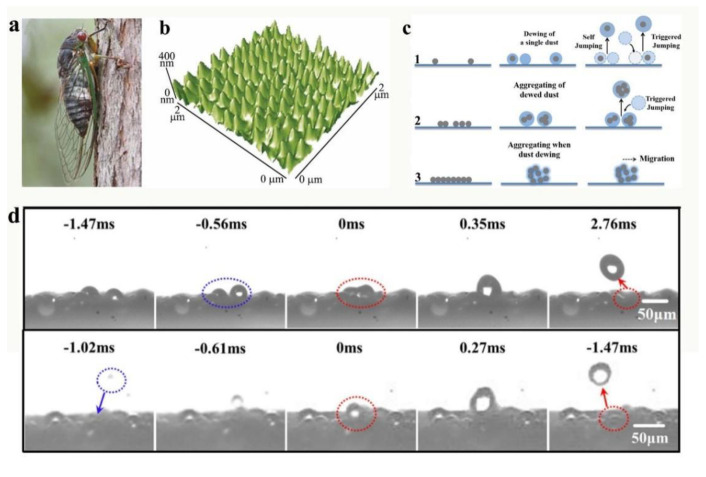
(**a**) The cicadas usually face the sky at rest; (**b**) the micro-/nanostructure of cicada wings, characterized by atomic force microscopy, reproduced with permission from Ref. [162]. (**c**) Three different mechanisms for removing dust particles during the condensation; (**d**) droplets on the coating surface exhibit both active and triggered jump motions, reproduced with permission from Ref. [163].

Figure 13a is a photograph of a cicada. The wing surface is distributed with many micro-/nanostructures and has hydrophobic wax layers, and thus has superhydrophobic properties (Figure 13b). When the condensation is formed, it can jump and remove the dust particles from the wings. Inspired by cicada wings, Zhan et al. [163] prepared a superhydrophobic coating with dewdrop self-bouncing function. Experimental results found that smaller dust particles are easily removed by bouncing dewdrops. The closer particles first fuse together and then jump apart. Large particles are not easily removed and therefore take longer to condense (Figure 13c). As illustrated in Figure 13d, there are two types of jumping motions: the first one lacks the intervention of external forces, where neighboring condensate droplets combine and bounce themselves. The second type is caused by a falling droplet touching a stationary droplet. After the condensate is formed, it will adsorb the dust particles on the glass surface and form a dust-carrying water droplet. Because it is a superhydrophobic surface, the contact area between the water droplet and the glass is small, and when adjacent water droplets are combined or triggered, the large amount of energy released will allow the water droplet to overcome the van der Waals and capillary forces, thus ejecting from the glass surface and carrying away the dust particles [164,165,166]. This process of nucleation, growth, fusion, and bouncing can continue repeatedly, during which most of the dust particles on the glass surface can be carried away.

## 5. Summary and Future Prospective

This paper summarizes the application of low van der Waals force surfaces, anti-static surfaces, and superhydrophobic surfaces for anti-dust applications on glass surfaces in three aspects: micro-/nanostructures, surface energy, and surface sheet resistance. Each easy-cleaning method has its unique advantages and application sites. In some dry areas with little rainfall, superhydrophobic easy-cleaning surfaces that rely on water for dust removal are of much less value, while low van der Waals force surfaces prepared by constructing micro-/nanostructures or reducing surface energy can effectively reduce dust deposition. The dust deposition on glass coated with hydrophobic silica sol, coated with SiO_2_ nanoparticles, or coated with both silica sol and SiO_2_ nanoparticles was 51.4%, 38.6%, and 36.1% of that of bare glass, respectively, which indicates that either the micro-/nanostructure or the low surface free energy can decrease dust deposition, and the combination of both is more effective in repelling dust particles. In desert areas where it hardly rains, large particles of dust on the glass surface can only be removed by wind. For removal of small dust particles, coatings that allow water dew to bounce back on its own play an important role. The self-bounce of dew condensation can clean the coated surface well, with an average light transmission rate of over 91%. However, the uncoated surface remained virtually unchanged, with a lot of dust particles still on the surface, and light transmission was below 80%. In rainy areas, superhydrophobic surfaces play a significant role in dust cleaning. It was reported that there was a 51% loss in output power when dust particles were deposited on the PV panel surface. With a superhydrophobic coating, 90% of the solar cell efficiency was restored. Anti-static surfaces only work in dry environments because in wet environments capillary forces play a major role and there is little electrostatic force. Although superhydrophobic coatings have a good performance in preventing dust particle accumulation, most superhydrophobic coatings reduce the substrate transparency. Micro- and nanostructures are required to obtain superhydrophobicity, but unfortunately, the structured surfaces lead to reduced transparency due to light scattering. Moreover, most of the superhydrophobic coatings are not durable due to the fact that the surface micro- and nanostructures can be easily damaged and thus lose their superhydrophobic properties. For the anti-static coatings, conductivity and transparency are also issues to be addressed as most of the conductive fillers would affect the transparency.

For glass products, light transmission and durability are the two most important factors. Light transmission is influenced by the surface roughness and structures. Most of the current coatings reduce the light transmission of the glass, hindering their applications. Coating durability is also a pending issue, as the easy-cleaning performance decreases significantly with time. Environmental factors, such as mechanical wear and acid rain corrosion, can damage the microstructures of the coating. Therefore, research needs to be carried out to develop coatings with long-lasting and transparent easy-cleaning properties.

At present, research on anti-dust surfaces mainly focuses on a single aspect, such as superhydrophobic surfaces, low van der Waals force surfaces, or anti-static surfaces. It is difficult for a single function anti-dust surface to cope with the complex and changeable external environment. We envision that future research will be focused on developing transparent and durable surfaces with multiple dust-proof functions to cope with the complex natural environment.

Exceptional easy-cleaning properties can substantially reduce the labor required to clean glass surfaces. In this paper, we have provided a comprehensive and in-depth review of the principles, methods and materials used for constructing anti-dust and easy-cleaning surfaces. It has been shown that in a dry environment, the main adhesion forces between dust particles and glass surfaces are van der Waals and electrostatic forces. In a humid environment, capillary forces account for 98% of the total adhesion forces. Most of the dust is electrically charged and is thus easily adsorbed.

We illustrate the dust-removal processes on anti-dust and easy-cleaning surfaces. Superhydrophobic surfaces remove dust particles using the rolling of water droplets and the self-bouncing of dew water. Anti-static surfaces are prepared by the addition of conductive nanomaterials to reduce the surface sheet resistance, leading to reduced dust particle adsorption. The analysis of advantages, disadvantages and applications of each method is provided.

For future research, focus will be in creating transparent and durable surfaces with multiple dust-proof functions to cope with the complex operating environments.

## Figures and Tables

**Table 1:** Dust particle composition in different areas.

Author	Location	Chemical Composition of Dust Particles	Ref.
Dhaouadi	United Arab Emirates	CaSO_4_, SiO_2_, Mg_2_(SiO_4_), Ca(CO_3_), Fe_2_O_3_, Ca_2_Mg(Si_2_O_7_),	[53]
Lu	China	SiO_2_, Al_2_O_3_, Fe_2_O_3_, CaO, K_2_O	[5]
Wu	China	SiO_2_, CaCO_3_, NaAlSi_3_O_8_	[54]
Al-Dousari	Kuwait	Quartz, Carbonates, Feldspars, Clay	[55]
Gholami	Iran	SiO_2_, CaO, Al_2_O_3_, Fe_2_O_3_, MgO, K_2_O, TiO_2_, SO_3_, MnO_2_, Cr_2_O_3_, SrO and NiO.	[56]
Hachicha	UAE	SiO_2_, CaO, Fe_2_O_3_, MgO, Al_2_O_3_	[57]

**Table 2:** Summary of methods for constructing superhydrophobicity.

Methods	Low Surface Energy Materials	Contact Angle (°)	Rolling Angle (°)	Light Transmittance (%)	Ref
Laser machining	1H, 1H, 2H, 2H-perfluorooctyltrichlorosilane	~180	-	-	[129]
	1H, 1H, 2H, 2H-perfluorodecyltriethoxysilane	161	2	92	[77]
Plasma etching	1H, 1H, 2H, 2H-perfluorooctyltrichlorosilane	~150	-	-	[130]
	perfluorooctyl triethoxysilane	166	-	-	[131]
Template transfer technology	Polymeric Methyl Methacrylate	152	3	-	[40]
	polydimethylsiloxane	152	4	93	[132]
Electrospinning	Trichlorosilane	158	-	-	[133]
	(Tridecafluoro-1,1,2,2-tetrahydrooctyl)-1-trichlorosilane	161	1	85	[134]
Sol-gel method	Triethoxymethylsilane	164	5	91.13	[135]
	Triethoxy (3,3,4,4,5,5,6,6,7,7,8,8,8-tridecafluorooctyl) silane	160	0	90	[136]
Acid etching	methyltrichlorosilane	154	3	-	[137]
	1H, 1H, 2H, 2H-perfluorodecyltriethoxysilane	170	2	-	[138]
RF magnetron sputtering	hexadecyltrimethoxysilane	155	-	-	[139]
	hexadecyltrimethoxysilane	169	1	-	[140]
AACVD	Polytetrafluoroethylene	168	1	90	[141]
	Polydimethylsiloxane	160	1	80	[142]

## Data Availability

Not applicable.
